# Programmed death ligand 1 expression in diffuse large B cell lymphoma: correlation with clinicopathological prognostic factors

**DOI:** 10.1186/s43046-023-00171-6

**Published:** 2023-05-08

**Authors:** Eman Mohamad Ibrahim, Sherine Refat, Shaimaa El-Ashwah, Maryan Waheeb Fahmi, Afaf Taha Ibrahiem

**Affiliations:** 1 Pathology Department, Faculty of Medicine, Mansoura University, Mansoura, 35516 Egypt; 2 Clinical Hematology Unit, Internal Medicine Department, Faculty of Medicine, Mansoura University, Mansoura, 35516 Egypt; 3 Medical Oncology Unit, Internal Medicine Department, Faculty of Medicine, Mansoura University, Mansoura, 35516 Egypt

**Keywords:** NHL, DLBCL, PD1, PD-L1, Disease-free survival, Prognosis

## Abstract

**Background:**

The prognostic value of the level of programmed death ligand 1 (PD-L1) expression in non-Hodgkin lymphoma (NHL) is still debatable. This study examined the effect of the level of PD-L1 expression on the clinicopathological characteristics and prognosis of diffuse large B cell lymphoma (DLBCL).

**Methods:**

A retrospective study was conducted on formalin-fixed paraffin-embedded tissue blocks of one hundred de novo DLBCL patients diagnosed from 2013 to 2016. PD-L1 expression was defined by a modified Combined-Positive Score (CPS) and their medical records were reviewed to collect their clinical, laboratory and radiological data, treatment, and outcome.

**Results:**

The included patients were aged from 23 to 85 years and treated by rituximab- cyclophosphamide, doxorubicin, oncovin, prednisone (R-CHOP); 49% were males; 85% of the cases were presented at Ann Arbor stages III, IV; 33% of patients were seropositive for HCV and 87% of cases were presented with intermediate and high IPI. All included cases expressed PD-L1 using modified CPS. 27% of patients showed low PD-L1 expression (≥ 5% to < 50% of total tumor cellularity) while 73% of patients showed high PD-L1expression (≥ 50% of total tumor cellularity). High PD-L1 expression is statistically correlated with advanced stage (*p* 0.01), high IPI score (*p* 0.017), high incidence of stationary and progressive disease (*p* 0.002) and high incidence of relapse (*p* value 0.01). Five-year disease-free survival (DFS) was 29% for patients with high PD-L1 expression compared with 84.8% for patients with low PD-L1 expression (*p* 0.001).

**Conclusions:**

This study suggests that high PD-L1 expression in DLBCL is associated with aggressive clinicopathological features and a decreased response to R-CHOP. The level of PD-L1 expression could be an independent predictor of DFS of DLBCL. More research is mandatory to standardize the cutoff value and scoring methods.

## Background

The incidence of non-Hodgkin lymphoma (NHL) has shown a dramatic increase over the past few decades. Diffuse large B cell lymphoma (DLBCL) represents the common type of NHL [1]. In Egypt, NHL was ranked the fifth most frequent malignancy in both genders [2]. The Ann Arbor staging system and the International Prognostic Index (IPI) are commonly used as prognostic factors [3]. However, the prognosis varies among the different histological types of NHL. The prognosis also differs among patients with DLBCL despite the same treatment strategy due to variable clinical features and molecular alternations. Therefore, the identification of new biomarkers could accurately predict the prognosis has great clinical and therapeutic implications [4].

Recently immunotherapy has enriched the landscape of cancer therapeutics. Immune checkpoint blockage with antibodies against programmed cell death protein 1 (PD-1), programmed cell death ligand 1 (PD-L1), and also cytotoxic T-lymphocyte antigen (CTLA-4) has produced hopeful results in various cancers [5].

PD-1, also referred to as CD279, is a receptor that is expressed on B lymphocytes (B cells), activated T lymphocytes (T cells), dendritic cells, macrophages, natural killer cells (NK cells), and monocytes [6]. PD-1 interacts with the PD-L1; also known as CD274 and PD-L2; also known as CD273, which lead to inhibition of the immune response. Both hematopoietic and non-hematopoietic cells exhibit high levels of PD-L1 expression. PD-L1 is constitutively expressed on T cells, B cells, antigen-presenting cells (APCs) and further up-regulated upon their activation. Comparatively fewer cell types express PD-L2 than PD-L1, mainly on APCs [7].

PD-L1 is expressed on malignant cells and tumor-infiltrating non-malignant stromal cells [8]. Recently, the impact of PD-L1 expression on the prognosis of various cancers has attracted the interest of many researchers because PD-1/PD-L1-mediates the tumor immune escapes [6].

The prognostic value of PD-L1 expression in DLBCL remains controversial. Some studies have proposed that the expression of PD-L1 is correlated with poor prognosis and could represent a valuable therapeutic target [8–10]. Kwon et al. found that positive PD-L1 expression carried no prognostic value, or correlated with favorable prognoses than those without [11].

In this study, we assessed the level of expression of PD-L1 in DLBCL using modified CPS and correlated these levels with other clinicopathological features and patients’ outcome.

## Methods

This retrospective study was performed on formalin-fixed paraffin embedded tissue from archives from the pathology laboratory of our oncology center. One hundred patients were included in the period from 2013 to 2016. All patients were diagnosed with de novo DLBCL and underwent excisional or incisional biopsies. Fine needle aspiration cytology specimens and core biopsies were excluded from the study. The medical records were reviewed to collect their clinical, laboratory and radiological data, treatment, and outcome. The achieved response was classified according to the Lugano classification [12].

The Institutional Review Board of our faculty reviewed and approved this study (IRB code: R.20.10.1038). The authors declare that the guidelines of the World Medical Association Declaration of Helsinki were followed.

Histopathological features were reviewed from H&E-stained slides. Cases were assigned as centroblastic, immunoblastic, and anaplastic. Immunohistochemistry for CD20, CD3, CD10, BCL-6, MUM-1, BCL-2, P53, and C-MYC was done through DAKO Autostainer Link 48. The cases were assigned as germinal/non-germinal center cells of origin using the Hans algorithm according to the 2017 WHO classification of hematopoietic and lymphoid tumors [13]. Tissue microarray blocks were constructed. Manual immunohistochemical staining for PD-L1 was done using polyclonal anti-PD-L1 antibody–Biospes–Catalog # YPA1637. Immunohistochemical study was conducted according to manufacturer instructions with appropriate positive and negative controls.

Here, PD-L1 expression was defined by modified Combined-Positive Score (CPS). In contrast to solid tumors, it was difficult to distinguish lymphoma cells from immune cells in DLBCL. For easier interpretation, we used the modified CPS method to assess the level of PD‐L1 expression as follows: the percentage of positive lymphoma cells and immune cells/total tumor tissue cellularity [14].

The cut off used in this study was 5% of the cellularity expressing cytoplasmic and or membranous PD-L1. Positive cases were considered as low (≥ 5 to < 50% of cells) or high expression (≥ 50% of cells) [15, 16].

P53 over expression was defined as strong nuclear expression of more than 30% of tumor cells [17]. BCL-2 was considered positive if ≥ 50% of the tumor cells showed cytoplasmic staining. C-MYC was considered positive if ≥ 40% of the tumor cells showed nuclear staining [13]. Double expressor (DE) cases were positive for BCL-2 and C-MYC. Triple expressor (TE) cases are positive for BCL-6 in addition [18].

### Statistical analysis

Data were analyzed using IBM SPSS Statistics for Windows, Version 22.0. Armonk, NY: IBM Corp. Qualitative data were defined as number and percentage. Quantitative data were defined as mean ± standard deviation for parametric data after testing normality using Kolmogrov-Smirnov test.

### Data analysis

- Chi-square test or Fisher’s exact test for comparison of 2 or more groups was used for qualitative data.
- Disease-free survival (DFS) was measured, in months, since date of complete response to the date of death, relapse, or the last follow-up visit. Overall survival (OS) was measured, in months, from the date of initial diagnosis to the date of death or the last follow-up visit. Survival data were estimated using the Kaplan–Meier curve method and the log-rank test was used for comparison.
- Cox regression analysis of factors potentially related to survival was performed to identify which independent factors might jointly have a significant influence on survival
- The *p* value is considered significant if < 0.05 at confidence interval 95%.

## Results

### Clinicopathological characteristics of the cases being studied

As shown in Table 1, the study included 100 cases of de novo DLBCL, the median age of studied cases was 59 years (ranged from 23 to 85 years), 49% were males, 33% were seropositive for HCV. Serum LDH was elevated in 79% of patients, 44% of cases had B symptoms. Bone Marrow was infiltrated in 20% of cases. Extra-nodal involvement was found in 26% of cases. 85% of cases were presented at an advanced stage (Ann Arbor stages III, IV). Eighty-seven percent of cases were presented with intermediate and high IPI.

**Table 1:** Patients’ characteristics

**Variables**	**No**	**%**
**Gender**		
Male	49	49%
Female	51	51%
**Age/years**		
≤ 60	52	52.0
> 60	48	48.0
**HCV seropositivity**		
Yes	33	33%
No	67	67%
**Serum LDH**		
Normal	21	21%
Elevated	79	79%
**B symptoms**		
No	56	56%
Yes	44	44%
**Involved sites**		
Nodal	74	74%
Extra-nodal	26	26%
**BM infiltration**		
No	73	73%
Yes	20	20%
Biopsy not done	7	7%
**Ann Arbor stage**		
I and II	15	15%
III and IV	85	85%
**IPI risk**		
Low	13	13%
Low intermediate	34	34%
High intermediate	33	33%
High	20	20%
**Histological type**		
Centroblastic	72	72%
Immunoblastic	19	19%
Anaplastic	9	9%
**Hans classification**		
GC	22	22%
Non-GC	78	78%
**P53 expression (*N* = 85)**		
No over expression	66	77.6%
Over expression	19	22.4%
**Double expressor**		
No	72	72%
Yes	28	28%
**Triple expressor**		
No	92	92%
Yes	8	8%
**PD-L1 expression**		
Low	27	27%
High	73	73%
**Treatment response**		
CR	42	42%
PR	11	11%
SD	3	3%
PD	44	44%
**Relapse after CR (*N* = 42)**		
No	28	67%
Yes	14	33%

Centroblastic variant was the commonest histological subtype, representing 72% of cases. According to Hans classifier, 78% of cases were of non-germinal center cell of origin. Over expression of P53 was noticed in 19 cases. Twenty-eight patients were double expresser and 8 patients were triple expresser.

**Fig. 1:**
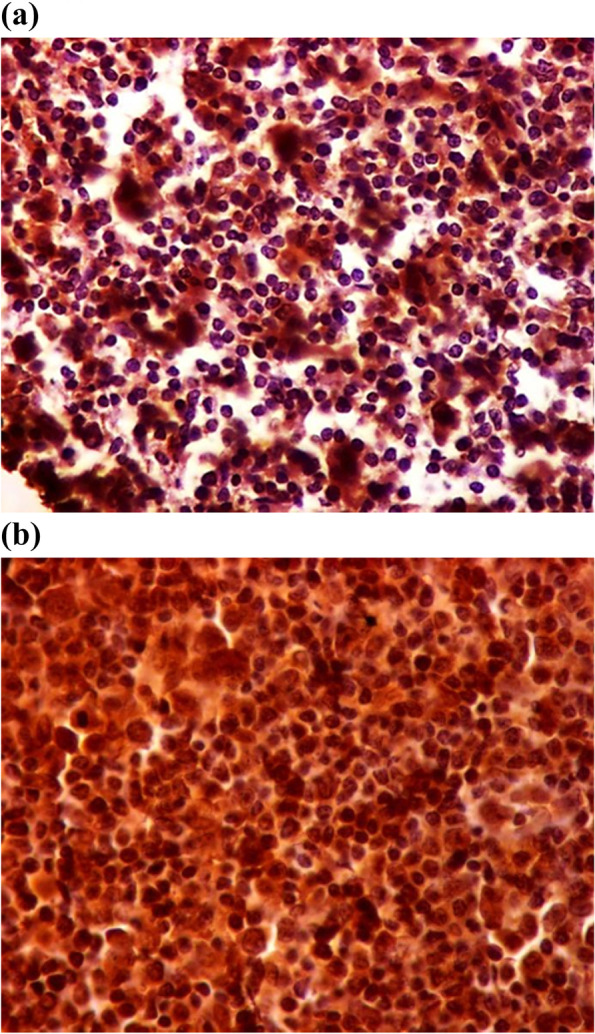
Immunohistochemical expression of PD-L1 in DLBCL: photomicrograph **a** shows low PD-L1 expression. Photomicrograph **b** shows high PD-L 1 expression. PD-L1 is expressed in the cytoplasm and cell membrane in lymphoma cells as well as the non-lymphoma cells using modified combined positive score

All included patients were treated by rituximab-cyclophosphamide, doxorubicin, oncovin, prednisone (R-CHOP protocol). Regarding treatment response; 42 patients achieved CR, 11 patients achieved PR, 3 patients had SD, and 44 patients showed disease progression. PD-L1 expression was detected in all included cases; 27 patients showed low expression; 73 patients showed high expression, as illustrated in Fig. 1.

### PD-L1 expression and clinic-pathological characteristics

High PD-L1 expression is statistically correlated with advanced stage (*p* 0.01), high IPI score (*p* 0.017), high incidence of stationary and progressive disease (*p* 0.002) and a high incidence of relapse (*p* value 0.01). Twenty patients of the thirty-three HCV seropositive DLBCL patients had high PD-L1 expression (*p* value 0.05).

No statistical association was found between level of PD-L 1 expression and gender (*p* 0.73), age (*p* 0.18), serum LDH (*p* 0.9), B symptoms (*p* 0.39), involved sites (*p* 0.3), bone marrow infiltration (*p* 0.76), histological types (*p* 0.124), Hans classification (*p* 0.56), P53 over expression (*p* 0.72), double expresser cases (*p* 0.07), triple expresser cases (*p* 0.67) as illustrated in Table 2.

**Table 2:** The association of PD-L1 expression with clinicopathological features

**Variables**	PD-L1 low expression *N* = 27(27%)	PD-L1 High expression *N* = 73(73%)	Test of significance	*P* value
**Gender**				
Male	14 (51.9%)	35 (47.9%)	0.12	0.73
Female	13 (48.1%)	38 (52.1%)		
**Age/years**				
≤ 60	17 (63%)	35 (47.9%)	1.78	0.18
> 60	10 (37%)	38 (52.1%)		
**HCV seropositivity**				
Yes	13 (48.1%)	20 (27.4%)	3.8	0.05
No	14 (51.9%)	53 (72.9%)		
**Serum LDH**				
Normal	6 (22.2%)	15 (20.5%)	0.03	0.9
Elevated	21(77.8%)	58 (79.5%)		
**B symptoms**				
No	17 (63%)	39 (53.4%	0.73	0.39
Yes	10 (37%)	34 (46.6%)		
**Involved sites**				
Nodal	18 (66.7%)	56 (76.7%)	1.03	0.3
Extra-nodal	9 (33.3%)	17 (23.3%)		
**BM infiltration (*N*** = **93)**				
No	15 (75%)	58 (79.5%)	0.18	0.76
Yes	5 (25%)	15 (20.5%)		
**Ann Arbor** s**tage**				
I and II	8 (29.6%)	7 (9.6%)	6.2	0.01*
III and IV	19 (70.4%)	66 (90.4%)		
**IPI risk**				
Low and low intermediate	18 (66.7%)	29 (39.7%)	5.74	0.017*
High intermediate and high	9 (33.3%)	44 (60.3%)		
**Histological type**				
Centroblastic	21 (77.8%)	51 (69.9%)	4.17	0.124
Immunoblastic	2 (7.4%)	17 (23.3%)		
Anaplastic	4 (14.8%)	5 (6.8%)		
**Hans classification**				
GC	7 (25.9%)	15 (20.5%)	0.33	0.56
Non-GC	20 (74.1%)	58 (79.5%)		
**P53 expression (*N* = 85)**				
No over expression	17 (81%)	49 (76.6%)	0.191	0.77
Over expression	4 (19%)	15 (23.4%)		
**Double expresser**				
No	23 (85.2%)	49 (67.1%)	3.19	0.07
Yes	4 (14.8%)	24 (32.9%)		
**Triple expresser**				
No	26 (96.3%)	66 (90.4%)	0.9	0.67
Yes	1 (3.7%)	7 (9.6%)		
**Treatment response**				
CR	18 (66.7%)	24 (32.9%)	14.9	0.002*
PR	3 (11.1%)	8 (11%)		
SD	2 (7.4%)	1 (1.4%)		
PD	4 (14.8)	40 (54.8%)		
**Relapse after CR**				
No	16 (88.9%)	12 (50%)	7	0.01*
Yes	2 (11.1%)	12 (50%)		

*χ*^2^ chi-square test, *MC* Monte Carlo test

^*^Significant* P* value < 0.05

### Factors affecting DFS and OS

**Fig. 2:**
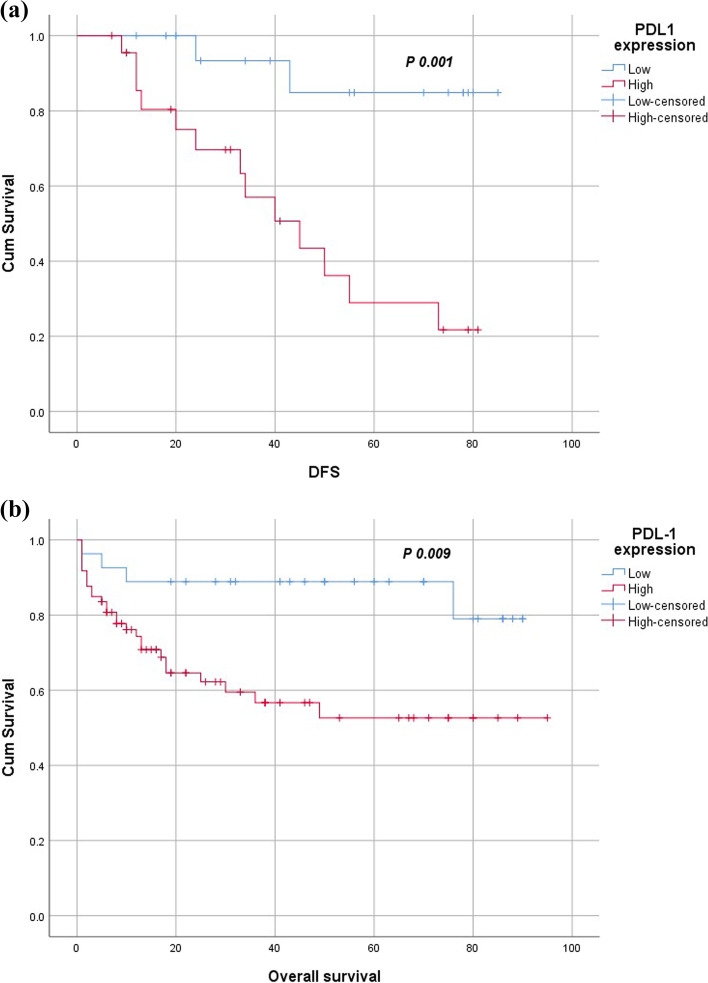
**a** Relation of disease free survival to PD-L1 expression level. **b** Relation of overall survival to PD-L1 expression level

The estimated one and 5-year DFS for the study cases were 92.2% and 58.5%, respectively. PD-L1 expression was a powerful prognostic factor with a 5-year DFS of 29% for patients with high PD-L1 expression compared with 84.8% for patients with low PD-L1 expression (*p* 0.001) (Table 3) (Fig. 2a). Cox regression analysis (Table 4) revealed that expression of PD-L1 is an independent predictor of DFS in DLBCL patients (*p* 0.028).

**Table 3:** Factors affecting disease free survival among studied cases

Variables	1 year	5 years	Test of significance	*P* value
Gender				
Male	95%	69.7%	2.917	0.088
Female	90%	36.4%		
Age				
≤ 60	90.9%	56.5%	0.033	0.856
> 60	93.8%	52.4%		
HCVseropositivity				
Negative	88.1%	54.6%	0.005	0.942
Positive	100%	53%		
B symptoms				
Absent	86.5%	52%	0.237	0.626
Present	100%	57.8%		
BM infiltration (*N* = 93)				
No	90.5%	54.3%	0.054	0.816
Yes	100%	50%		
Hans classification				
GC	100%	100%	3.704	0.054
Non-GC	90.6%	46.6%		
IPI risk				
Low and low intermediate	92%	61.9%	0.462	0.497
High intermediate and high	92.3%	38.7%		
P53 expression (*N* = 85)				
No over expression	96%	49.4%	0.04	0.841
Over expression	76.2%	50.8%		
Double expresser				
No	90.8%	56%	1.987	0.159
Yes	100%	50%		
Triple expresser				
No	92%	63.7%	1.129	0.288
Yes	–	–		
PD-L1 expression				
Low	100%	84.8%	10.806	0.001*
High	85.4%	29%		

Kaplan–Meier curve method and the log-rank test

**Table 4:** Cox regression multivariate analysis for prediction of disease-free survival

Predictors	β	SE	Wald	*P* value	Hazard ratio	95.0% CI for HR	
						Lower	Upper
Hans classification	11.440	280.863	0.002	0.968	92,984.738	0.000	1.096E + 244
PD-L1 expression	1.674	0.761	4.832	0.028	5.331	1.199	23.704

One and 5-year OS estimated for the studied cases were 78.4% and 65.1%, respectively. Statistically longer OS was observed in patients without bone marrow infiltration (*p* 0.02), low and low intermediate IPI risk (*p* 0.001) and low PD-L1 expression (*p* 0.009) (Table 5) (Fig. 2b). Cox regression analysis (Table 6) revealed that only IPI risk was an independent predictor for OS in DLBCL patients (*p* 0.028).

**Table 5:** Factors affecting overall survival among studied cases

Variables	1 year	5 years	Test of significance	*P* value
Gender				
Male	83.1%	71.4%	0.858	0.345
Female	73.8%	58.9%		
Age				
≤ 60	86.2%	71.4%	2.248	0. 134
> 60	70.1%	59.4%		
HCV seropositivity				
Negative	79.8%	70.1%	0.602	0.438
Positive	75.5%	56.2%		
B symptoms				
Absent	78.1%	70%	0.752	0.386
Present	78.8%	59.1%		
BM infiltration (*N* = 93)				
No	84.3%	71.6%	5.381	0.02*
Yes	70%	50.5%		
Hans classification				
GC	80.6%	67.4%	0.011	0.918
Non-GC	77.6%	64.8%		
IPI risk				
Low and low intermediate	93.5%	80.3%	10.65	0.001*
High intermediate and high	64.5%	51.2%		
P53 expression (*N* = 85)				
No over expression	78.6%	61.4%	0.139	0.71
Over expression	83.1%	75.5%		
Double expresser				
No	80.4%	69.8%	2.005	0.157
Yes	73.9%	52.3%		
Triple expresser				
No	78.7%	65.9%	0.471	0.493
Yes	75%	–		
PD-L1 expression				
Low	88.9%	88.9%	6.729	0.009*
High	74.3%	52.6%		

Kaplan–Meier curve method and the log-rank test

**Table 6:** Cox regression for prediction of overall survival

Predictors	β	SE	Wald	*P* value	Hazard ratio	95% CI HR	
						Lower	Upper
BMI	0.678	0.406	2.790	0.095	1.970	0.889	4.366
PD-L1 expression	0.905	0.561	2.603	0.107	2.472	0.823	7.420
IPI risk	1.004	0.458	4.799	0.028	2.729	1.111	6.702

## Discussion

DLBCL is a diverse disease as regards clinical, histopathological, immunohistochemical and genetic features. Patients with DLBCL do not show uniform response to the first line immunochemotherapy. This highlights the need for novel markers to accurately predict the response and prognosis and may be used as a tool for target therapy [19].

PD-1 is usually up-regulated in lymphoma cells. PD-L1and PD-L2 could be expressed by tumor cells and the surrounding tumor microenvironment. PD-1/PD-Ls interaction participates in immune escape and subsequent lymphomagenesis [9]. PD-L1 and PD-L2 were up regulated in malignant B-cell lymphomas through intracellular and extracellular mechanisms and, variations in the structure of the 3′-untranslated region of the PD-L1 gene affect PD-L1 expression [20, 21].

Regarding the hematological malignancies, pembrolizumab and nivolumab (monoclonal antibodies to PD-1 that block the interaction between PD-1 and its ligands, PDL1 and PDL-2) have been approved for treating relapsed and refractory classical Hodgkin lymphoma and primary mediastinal large B cell lymphoma to date [22–24].

Assessment of the level of PD-L1 expression in tumor tissue helps to define patients who are most likely to respond to treatment. In relapsed and refractory lymphoma, the over-expression of PD-L1 on the surface of tumor cells was associated with a better response to anti-PD-1 therapy [25].

Several difficulties are met during the assessment of PD-L1 expression. These encompassed the specificity of different clones of anti–PD-L1 antibodies for IHC and technical aspects including tissue fixation, processing and antigen retrieval [26]. Various commercially available PD-L1 IHC companion/complementary diagnostic assays are available. The companion diagnostic assay may define patient eligibility to anti-PD-L1 therapy. Validity, cut-off, and reporting show marked-variability among different platforms [27]. Regarding the cellular localization of PD-L1, difficult scoring systems are adopted. The tumor proportion score (TPS) measures the proportion of positive PD-L1 tumor cells among the total viable tumor cells. The combined proportion score (CPS) estimates the ratio of the overall positive cells (tumoral and non-tumoral) to the total number of viable tumor cells multiplied per 100. Modification of CPS relates the positive tumor cells and immune cells to the overall cellularity. While, the area filled by PD-L1-positive immune cells in relation to the whole tumor area is referred to as the immune cell score (IC). Furthermore, some studies included the intensity of staining in their scoring algorithms [28].

There is no consensus of what is the relevant cut-off that splits up positive from negative results. The cut-off for positive PD-L1 across different studies ranged from 1 to 50% [29].

Hawkes et al. published that PD-L1 is not commonly expressed in B cell NHL [30], Only 10–24% of DLBCL cases express PD-L1. Higher rates of PD-L1 expression have been detected in certain subtypes as EBV-associated DLBCL, T cell histiocyte-rich DLBCL, primary mediastinal LBCL, and activated B cell DLBCL [21, 31, 32].

Using modified CPS in this study, all included patients diagnosed with DLBCL expressed PD-L1; 27% patients showed low expression (≥ 1 to < 50% of cells) and 73% patients showed high expression (≥ 50% of cells).

Gravelle et al. published that approximately 20–30% of DLBCL expressed PD-L1 [9].

Unfortunately, EBV encoded RNA (EBER-ISH) or IHC for latent membrane protein (LMP) were not available in this retrospective study as EBV test was not routinely requested for NHL work up in our center. Chen et al. found that 100% of EBV-positive DLBCL expressed PD-L1 in tumor cells, as well as in the tumor microenvironment considering cut-off for PD-L1 positivity more than 5% of tumor cells with intensity level of 2 + or 3 + or PD-L1 positivity more than 20% of the tumor microenvironment (TME) with the level of staining intensity of 2 + or 3 + [31]. Kwon et al. found 61.1% of DLBCL expressed PD-L1 using 10% cut-off and included the intensity in scoring [11].

It is worth noting that 33% of our included patients were HCV sero-positive and HCV sero-positivity was associated with the level of PDL-1 expression (*p* 0.05). Abdellatif and Shiha found that PD-L1 expression in CD34 + hematopoietic stem cells was upregulated in chronic HCV infection [33]. Chen et al. found that PD-L1 was overexpressed on malignant cells and tumor infiltrating macrophages in virus-associated malignancies [31]. Mofrad et al. found that tumorigenic viruses can inhibit the anti-cancer immune system by several mechanisms; one of them is by overexpression of PD-1/PD-L1 [34].

We found no significant correlation between levels of PD-L1 expression and patients’ age, gender, serum LDH, B symptoms but the levels of PD-L1 expression were statistically associated with IPI score and poor prognosis. These results were consistent with that of Zhao et al. who meta-analyzed nine studies (five of them were DLBCL subtype) to assess the correlation between PD-L1 expression and clinicopathological characteristics and prognosis of NHL [35].

Dissimilar to our results, Zhao et al. found that PD-L1 expression in DLBCL was not associated with Ann Arbor stage (*p* 0.44) but they mentioned some limitations to the results of their meta-analysis including different sources PD-L1 antibodies, different cut-off values and a possible publication bias [35]. Another meta-analysis was carried out by Zeng et al. on 12 studies of NHL (6 of them were DLBCL). Their results revealed that the overexpression of PD-L1 was associated with B symptoms, higher IPI score ≥ 3, and Ann Arbor stages (III and IV) as well as poor prognosis in the patients diagnosed with DLBCL [36].

Regarding the PD-L1 correlation with prognosis, high PD-L1 expression was associated with a dismal outcome [35–37]. Qiu et al. found that positive PD-L1 expression is statistically associated with shorter PFS and OS in DLBCL and its prognostic significance increased significantly when the cutoff value was ≥ 30% [37]. The results of meta-analysis conducted by Geng et al. indicate that PD-L1 expression detected by immunohistochemistry was a promising marker for the identification of patients who may benefit from blocking PD-1/PD-L1 by immunotherapy [25]. Smith et al. found that the CR rate and 2-year PFS were improved when pembrolizumab was added to R-CHOP in previously untreated PD-L1 expressing DLBCL [38].

## Conclusions

PDL-1 overexpression in DLBCL is associated with aggressive clinicopathological features and a lower response to standard RCHOP and worse prognosis. PD-L1 inhibitors may be promising in the initial treatment regimen for those patients.

However, more prospective multicentric research on larger study population is mandatory to validate and standardize the cut-off, scoring method, and site of expression.

## Data Availability

All the clinical, radiological, and pathological data used in this manuscript is available on Mansoura University medical system (Ibn Sina Hospital management system). http://srv137.mans.edu.eg/mus/newSystem/. IHC results for PDL1 are available from Assistant Professors of Pathology Dr. Sherine Refaat and Afaf Taha Ibrahiem and Lecturer of pathology Dr. Eman Mohamad Ibrahim on reasonable request.

## Abbreviations

APC: Antigen-presenting cells
B cells: B lymphocytes
BCL2: B cell lymphoma 2
CD: Cluster differentiation
CTLA: Cytotoxic T-lymphocyte antigen
DLBCL: Diffuse large B cell lymphoma
DFS: Disease-free survival
DE: Double expressor
EBER-ISH: EBV encoded RNA in situ hybridization
EBV: Epstein-Barr virus
HCV: Hepatitis C virus
IC: Immune cells
IPI: International Prognostic Index
LDH: Lactate dehydrogenase
LMP: Latent membrane protein
mCPS: Modified Combined-Positive Score
MUM-1: Multiple myeloma 1
NK cells: Natural killer cells
NHL: Non-Hodgkin lymphoma
OS: Overall survival
PD-1: Programmed death protein 1
PD-L1: Programmed death ligand 1
R-CHOP: Rituximab-cyclophosphamide, doxorubicin, oncovin, prednisone
T cells: T lymphocytes
TE: Triple expressor
TME: Tumor microenvironment
